# Understanding Environmental Health Issues to Inform Community Engagement in the Central Midwest

**DOI:** 10.21203/rs.3.rs-7410407/v1

**Published:** 2025-09-23

**Authors:** Madeline A. McCabe, Jacqueline Curnick, Tabitha Kuehn, Alithea Zorn, Brandi Janssen

**Affiliations:** Johnson County, Iowa; University of Iowa; University of Iowa; University of Iowa; University of Iowa

## Abstract

**Context:**

This report shares the results of a novel environmental health needs assessment designed by the Environmental Health Sciences Research Center in collaboration with our community partners for the four-state region of Iowa (IA), Nebraska (NE), Missouri (MO), and Kansas (KS).

**Objective:**

The goal of the needs assessment was to understand considerations about health and the environment among people living in the central Midwest states of Iowa, Nebraska, Kansas and Missouri, including their concerns, what they see as solutions, and what and how populations are vulnerable. These findings will inform future community engagement related to environmental health.

**Design:**

The assessment used a mixed-methods approach which included an online survey that collected quantitative and qualitative data and three focus groups with key populations of interest.

**Setting:**

University of Iowa Environmental Health Sciences Research Center in collaboration with our community partners for the four-state region of IA, NE, MI, and KS and focus groups in eastern IA.

**Participants:**

Over 400 participants from four states (IA, NE, KS, and MO)

**Main Outcome Measure(s):**

Understanding residents’ perceptions of the connections between environment and health in the 4-state area and identifying notable differences between rural, urban, and suburban populations

**Result:**

Top issues of concern were water quality, outdoor air quality, climate change, chemical contaminants, and natural disasters. Respondents identified better policies, access to medical services, and financial resources as key components of reducing personal risk to environmental health issues. The focus group sessions highlighted concerns about housing, social capital, and the need for trusted sources of information.

**Conclusions:**

These data suggest a holistic approach to environmental health topics, interest in policy interventions in rural communities, and potential for engagement with clinicians.

## Introduction

Communities in the central Midwestern states of Iowa, Nebraska, Kansas, and Missouri (EPA Region 7) frequently experience extreme weather events exacerbated by climate change [1, 2] as well as environmental health exposures that stem from agricultural activities [3], housing deficiencies [4], and naturally occurring contaminants such as radon and arsenic [5, 6]. The Community Engagement Core (CEC) of the Environmental Health Sciences Research Center (EHSRC) based at the University of Iowa conducted a region-wide needs assessment to better understand residents’ concerns. The findings will be used to tailor community engagement activities to align with resident perspectives on environmental health issues and support the solutions they identify.

## Background

The Environmental Health Science Research Center (EHSRC) at the University of Iowa, builds capacity in environmental health research. The goal of the EHSRC Community Engagement Core (CEC) is to address rural health disparities and enhance the community engagement of EHSRC research through meaningful collaboration with rural audiences. Community engaged research provides the necessary local context to understand environmental health issues. In a 2006 study, Larsson et.al. explored how rural community leaders frame, interpret, and give meaning to environmental health issues affecting their constituents and communities [7]. Guiding their inquiry was the principle of upstream thinking, which considers the social, economic, and environmental origins of health issues that manifest at the population level [8]. To organize their inquiry, Larsson et al. created an interview guide based on Dixon and Dixon’s [9] integrative environmental health framework, which posits that environmental health information can be conceptualized into four domains:

- Physiological (environmental agents and respective toxicodynamics – what are the problems?)
- Vulnerability (attributes that lead to increased susceptibility to environmental health risks – Who is affected by the problem?)
- Epistemological (how environmental risk information comes to be known and understood – How do people know about this problem?)
- Health protection (actions to reduce risks – What is done or should be done about the problem?)

Our study adapts this framework to contextualize environmental health issues in EPA Region 7.

Perspectives on environmental health issues and appropriate solutions extend across ruralities and partisan lines. In a 2020 study of rural attitudes on climate change in the upper Midwest, Bonnie and colleagues concluded that perceptions of weather change and vulnerability to climate impacts depends on partisanship, with rural Republicans reporting experiencing less extreme weather changes than rural Democrats. They also found that rural voters were less supportive of government oversight of the environment than their urban/suburban counterparts. Attitudes toward government oversight were also correlated with views on climate change among both urban and rural voters [10]. This work informed the analysis of our findings by rurality and interest in policy solutions.

## Methods

This mixed-methods needs assessment included a survey with both quantitative and qualitative responses followed by three focus group sessions. All study procedures were reviewed by the University of Iowa Institutional Review Board (IRB-01, #202308027), which determined that the project did not meet the regulatory definition of human subjects research because the activity is a needs assessment focused on a specific midwestern area and not intended to be generalizable.

### Survey Development

Our survey was developed to help the CEC respond to the environmental health concerns expressed by residents in our four-state region of Iowa, Nebraska, Missouri, and Kansas. Survey development began with a guided discussion with the Stakeholder Advisory Board (SAB) of the EHSRC. This group includes representation from county public health offices, environmental advocacy groups, citizen activists, medical providers, and other public health professionals. The SAB helped generate topic areas for survey respondents to prioritize, audiences for distribution, and questions for qualitative data collection. In addition, we consulted affiliated scientists in the EHSRC which includes environmental advocates, county environmental health specialists, physicians, toxicologists, and environmental health researchers. Finally, available literature informed the survey development. For example, in Robson & Schneider’s [11] study of rural health care providers’ beliefs about environmental health issues challenging their communities, emergent issues included water pollution, pesticide misuse, and soil erosion. We developed the questions on the survey instrument based on a synthesis of these sources and organized it based on the four domains described above developed by Larsson, et al.[7].

We pilot tested the survey among our own professional networks to ensure clarity in the questions and to invite feedback from these first participants about question clarity, flow, and survey length (N = 49). We also collected responses through SAB networks and EHSRC member networks (N = 93). This dataset was analyzed for program purposes only and is not included in the analysis here because of different distribution methods and populations.

### Survey Distribution

The Iowa Social Science Research Center (ISRC) managed survey distribution. The ISRC contracted with a web panel vendor, Qualtrics Research Services (QRS), to field the survey to a demographically representative sample of 400 respondents, with 100 respondents from each of the four states. Adults 18 years and older residing in these states were recruited to participate.

### Focus groups

Three focus groups were conducted to collect in-depth qualitative data. Each focus group aimed to better understand the perspectives of a particular demographic: young adults, older rural adults, and immigrants or people of color. Focus group discussion guides were developed based on the survey questions and organized by the four domains outlined in Larsson, et al. (2006). Participants were invited to brainstorm environmental health issues that affect them, reflect on community members who are most vulnerable to environmental health issues, and describe entities able to solve environmental problems. Each focus group was led by two study authors, one who led the discussion and the other who took detailed notes and ensured the sessions stayed within a 90-minute timeframe. The focus groups were audio recorded using the Voice Memos application and transcribed using Amazon Business Suite. All focus group participants were compensated with $10 Amazon Gift cards for their time.

### Statistical Analysis

The quantitative approach was primarily descriptive. Mean and medians were calculated to summarize responses to survey questions. Chi-square tests were used to detect differences in survey responses, racial groups, and ruralities.

## Results

### Physiological Domain

The first domain, physiological, was introduced to survey respondents with the following statement: In this section, we will be asking about what you see as important environmental problems that can affect human health. Respondents were asked to rate how much they thought the environment influenced people’s health, indicate their level of concern about several environmental health issues, and select environmental health issues of concern in their home.

When asked, “On a scale of 1 to 5, where 1 is no effect and 5 is a significant effect, how much do you think the environment affects people’s health?” respondents’ average rating was 3.88, indicating they believe the environment moderately to significantly affects people’s health.

We asked respondents to report their level of concern, on a scale from 1–5, about a list of environmental health issues. Issues were randomized so that they were not listed in the same order for any participant. Table 1 reports average level of concern for each environmental health issue; respondents reported highest concern about water quality, outdoor air quality, chemical contaminants, and climate change. There was no significant difference observed in level of concern by rurality.

Analysis of qualitative responses identified eight related themes: political apathy, concern for the future, call for collective action, positive environmental action, issues are overstated, negative health impacts, related to agricultural/rural exposures, and social determinants of health. Table 2 presents the themes, number of responses, and exemplar quotations.

Focus group respondents added further nuance and complexity to these findings. In particular, they showed how the areas of concern are not discrete topics; rather, people consider cumulative and additive effects of multiple hazards. For example, one rural resident stated: “I would say in addition to water quality, air quality by pesticide uses, you know, sprays. We lived out in the country. I was mowing the lawn one day, had ear buds on, so I couldn’t hear what was going on around me, but a farmer was spraying, and I didn’t know it until I could taste it in my mouth. And the wind was blowing directly at me. So air quality also.” In this case, while the participant initially implicated pesticides as a water quality issue, they also considered how they affect air quality.

**Figure 1:**
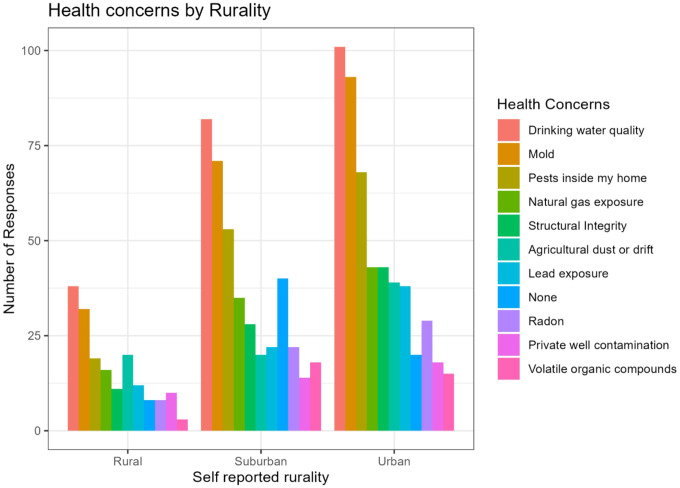
Health Concerns by Rurality

Respondents were also asked to reflect on their home environment, selecting all that apply from a list provided. Overall, the top three environmental health issues of concern in the home were Drinking Water Quality, Mold, and Pests Inside My Home. When analyzed by rurality, Drinking Water Quality and Mold were the most and second most common concern among all ruralities. Suburban and urban respondents reported Pests Inside the Home as the third concern, while rural respondents reported Agricultural Dust or Drift (Figure 1).

Participants in the focus group with mostly immigrants and people of color discussed the issue of pests in the home at length, describing several pest concerns, especially cockroaches and bedbugs. In addition, they described health impacts and identified responsible parties for these pests. For example, one responded noted, *“It’s just like a massive infestation of cockroaches and other insects that was harmful for my kids because one of the kids had asthma and, uh, at the same time, the property management had enough power for me that I was kind of locked in the system… the point in here is many of the families do not have this power. So, they really get stuck even if this is detrimental to the health of the kids.”*

### Vulnerability Domain

The second section of the survey was introduced with the following statement: In this section, we will ask about who you think is affected by environmental health issues. This section asked participants to reflect on how vulnerable they perceived themselves, as well as others in their communities.

When asked to rank themselves as more, less, or the same level of vulnerability as others most respondents viewed themselves as the same (70.4%) followed by 17.3% as more vulnerable and only 12.3% as less.

Children and senior citizens were identified as the most vulnerable to environmental health issues. When invited to comment, respondents noted that the children are especially vulnerable due to their immune systems and increased exposures to floors and lead dust. Senior citizens also carried the concerns of immune systems plus co-morbidities associated with age. Respondents also described social determinants of health as being related to environmental health issues; examples included homelessness and weather, income, access to medical care, veteran status, and discrimination. One individual also noted that “we are all vulnerable” to extreme weather and other climate events.

When analyzed by racial identity, the respondents from minoritized backgrounds were statistically more likely to identify racial or ethnic minorities as a vulnerable population (32.7%) than white respondents (19.9%). This same trend holds true for selecting immigrant populations as a vulnerable population (white: 20.3% and minoritized: 29.1% non-white, p= 0.01).

To understand how respondents conceptualized community-level vulnerabilities, we asked them to identify assets their communities had to prepare for a natural disaster. Respondents listed various physical infrastructure such as community buildings, resources and materials like sandbags and flood pumps, and communication systems. Respondents also identified leaders and public servants, organizations, and average citizens who work together to take care of each other.

Respondents were asked about preparedness for natural disasters on a Likert scale from 1–5, with 1 being the least prepared and 5 being the most prepared.

Overall, respondents view both their community (mean 3.01) and individual household (mean 2.98) as moderately prepared for a natural disaster. Among ruralities, there were non-significant but observable differences. Perceived community-level preparedness was lowest among rural participants (mean 2.81) and highest among suburban (mean 3.16); urban respondents fell in the middle (mean 2.93). For household preparedness, both rural and suburban respondents averaged 3.05. Thus, rural respondents may perceive their households to be more prepared than their communities, but suburbanites view their communities as more prepared than their individual households. Urban respondents rated their community preparedness (mean 2.93) as slightly higher than their household preparedness (mean 2.89).

### Epistemological Domain

The Epistemological domain was introduced with the statement: In this section, we will be asking about what and how you know about environmental health issues.

Overall, respondents considered themselves moderately knowledgeable about environmental health issues. Over half (62.9%) said that environmental health issues do not get enough attention, 30.5% said they receive the right amount of attention, and only 8.3% responded that environmental issues receive too much attention.

When seeking environmental health information, respondents consult their local health department, local media, state agencies, and federal agencies. The most common social media news source of environmental health information was Facebook, followed by YouTube and TikTok. These responses were consistent across rurality (urban, rural, and suburban).

In this domain, participants were also asked about their level of optimism and pessimism about the environment. Those who reported higher knowledge about environmental health issues also reported higher levels of optimism about the environment, while low levels of environmental health knowledge was correlated with higher levels with pessimism (χ^2^ = 64.1, p< 0.01). However, in a somewhat contradictory finding, people with higher self-reported knowledge reported higher levels of concern across several environmental health issues (Domain 1). This was most prevalent with climate change, indoor air quality, natural disasters, chemical contaminants, heavy metal exposure, and conservation.

### Health Protection Domain

The fourth domain, health protection, was introduced to survey respondents with the following statement: In this section, we will be asking about what you think is being done or should be done about environmental health issues. Respondents were asked to indicate how able individuals in their communities were to protect themselves, if they felt they would have help in the event of a natural disaster, individuals or organizations that are responsible for protecting residents, and services or resources that would be most beneficial to reduce personal risk.

When asked, “Do you feel you would have access to help if you experienced problems related to the environment (housing issue, natural disaster, flooding, extreme weather)?” most respondents (51.9% N=213) responded “yes;” 22.4% (N=92) responded “no,” and 27.1% (N=111) responded “I don’t know.”

The next question asked, “What individuals or groups are responsible for protecting residents from environmental health problems in our region?” Responses were open ended and thematically coded into the following domains: local leadership, state agencies, federal agencies, non-profits, and other groups. Notably, 67 respondents (16%) noted “government.” Other specific responses included mayor, police, FEMA, EPA, Red Cross, churches, food banks, lawyers, as well as “we each are,” and “we have a local emergency director who has many responsibilities including environment, health, natural disasters, etc.”

Respondents were asked, “which of the following would be the most beneficial to you in reducing your personal risk for environmental health issues?” They were restricted to selecting one of the following options, which were randomized so that they were not listed in the same order for each participant:

- Financial resources to make improvements in in housing or other personal risk factors
- Better policies at the local, state, or federal level to reduce the risk of environmental health issues
- More information about environmental health issues
- Better access to medical services/health screenings that could identify health problems caused by environmental issues
- Environmental sampling/monitoring in my community to identify health risks
- Changes in my workplace or work environment
- Other (please describe)

Overall, respondents prioritized better policies first, followed by better access to medical screenings, and then financial resources to make improvements. Perceived ways to reduce personal risk significantly differed by rurality (χ^2^= 22.5, p=0.03). Urban respondents reported better access to medical services as most important and suburban and rural respondents reported better policies.

**Figure 2:**
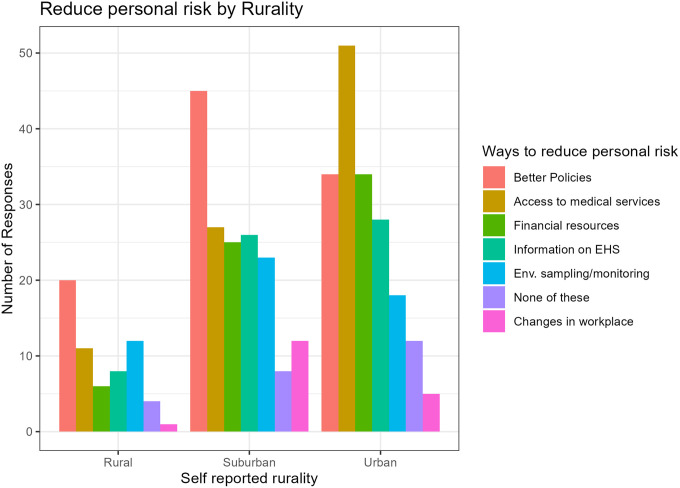
Reduce personal risk by rurality

As seen in Figure 2, urban communities reported access to medical services as the number one way while suburban and rural communities reported better policies.

Participants in the rural focus group elaborated on policy solutions, describing strategies that would hold polluters or emitters accountable, including direct action such as banning pesticide application on household lawns. Some participants expressed doubt that policy actions are feasible; one stated, “I don’t think that, at least the Iowa legislature, I don’t think they always respond to what the voters want.”

## Discussion

We conducted a multi-state needs assessment to understand environmental health issues in the Midwest using four domains – physiological, vulnerability, epistemological and health protection – to create more effective environmental health outreach and communication strategies. Our focus group discussions demonstrate how communities experience environmental health issues holistically because poor housing, pollution, and water quality issues often occur alongside each other. This is consistent with other studies; for example, Crighton and colleagues [12] investigated how new mothers perceive environmental health risks to their children. Even though researchers identified a wide range of environmental risk perceptions among participants, they found that any health-promoting action taken by the mothers was grounded in their daily sensory experiences of those issues. Similarly, White and Hall [13] found that participants identified environmental hazards in their community based on their direct experiences with the hazards. Together, these studies show that community members use their tangible, lived experiences to understand environmental health issues. This may contribute to why the discrete survey responses did not clearly differentiate levels of concern between different environmental health issues.

Our findings support intervention approaches that center community members’ lived experiences and local context to be effective [14]. Future interventions could employ an approach that reflects the reality of overlapping environmental health issues by organizing partners across several different sectors of the community (i.e., housing, transportation, emergency management, agriculture). This strategy supports comprehensive, community-centered interventions that may better resonate with communities.

We also sought to understand which groups were perceived to be most at risk to better develop programming for these communities and population groups. Our finding that children and elderly age populations are viewed as vulnerable is consistent with the literature. Risher et al. [15] examined how elderly populations are particularly impacted by environmental exposures for biological and medical reasons. Others have explored how children are susceptible to environmental hazards in the first 1000 days of life through exposures primarily in the home [16]. The authors also acknowledge non-environmental factors such as socio-economic status and emerging environmental issues as particularly relevant to these populations. Our finding that rural residents perceive their household preparedness to be higher than community level preparedness suggests that more needs to be done to strengthen rural community preparedness. Rahe et al. [17] examined community ties in rural development projects and found that financial resources and social capital are intertwined and should both be used to boost prosperity. Conversely, given that urban and suburban respondents viewed communities as more prepared than individual households, outreach efforts in these areas could be focused on increasing individual and household preparedness and confidence.

## Implications for policy and practice

One somewhat surprising finding was the interest in policy solutions from rural respondents. Rural communities have been seen as distrustful of regulatory governance [18], including in relation to environmental policy. Scholars have long characterized the rural distrust of environmental policy as a binary distinction in opposition to urban acceptance [19]. In addition, rural communities have high exposures to extractive industries as well as deep economic ties to these industries [20, 21]. As a result, rural residents observe or engage in environmentally detrimental practices and bear most of the associated physical, social, and material costs [22, 23]. Our finding that rural residents support policy intervention suggests an opportunity to identify acceptable and effective policy solutions for environmental health issues in rural areas. Further research focusing on factors that influence policy acceptability [24], perceived effects of policy [25], or overcoming structural barriers to policy change [26] may be fruitful next steps.

Urban residents showed interest in clinical solutions and increased access to medical services. Clinical solutions to environmental health issues are being explored. For example, physicians have shown to acknowledge the importance of discussing the health impacts of environmental exposures in workplaces and residences with patients. Despite urban residents being interested in these interventions and physicians valuing the importance of discussing these topics with patients, many physicians do not frequently ask their patients about environmental exposures, and most physicians have not received recent trainings on environmental exposures. Many physicians have knowledge gaps about environmental exposures, including PFAS which is a common contaminant in drinking water, a category that urban residents indicated as a top health concern [27]. To support access and frequency of discussing environmental health topics with clinicians for urban residents, developing environmental exposure training and resources for clinicians and integrating environmental exposures screenings at medical visits may be productive next steps.

## Conclusion

The Community Engagement Core (CEC) of the Environmental Health Sciences Research Center (EHSRC) based at the University of Iowa conducted a region-wide needs assessment to better understand residents’ concerns, knowledge, and ideas for intervention. We will use these findings to tailor community engagement activities to align with community perspectives on environmental health issues and support the solutions they identify. Survey respondents from Iowa, Nebraska, Kansas, and Missouri identified numerous environmental health challenges, as well as assets and needs within their local communities. These data will inform future environmental health education, outreach, and interventions in EPA region 7.

## Figures and Tables

**Table 1:** Environmental Health Issues Listed by Average Level of Concern

Issue	Mean	Median
**Water quality**	3.64	4
**Outdoor air quality**	3.53	4
**Climate change**	3.44	4
**Chemical contaminants**	3.39	4
**Natural disasters**	3.31	3
**Need for clean energy**	3.30	3
**Conservation**	3.25	3
**Indoor air quality**	3.19	3
**Soil health**	3.15	3
**Heavy metal exposure**	3.09	3

**Table 2:** Additional Comments about Environmental Health Issue Concerns

Theme	N	Example quotation
**Political Apathy**	16	“*My concern, and the reason I’m pessimistic about alleviating climate change issues, is that many politicians and governments don’t take the matter seriously. For example, a presidential candidate who won your state’s caucus has said that if he wins he’ll drill, drill, drill*.”
**Concern for the future**	15	“*I worry about future generations. How will they manage if we do not do something now each and every day. We need to conserve our energy and resources for future generations. We must be careful and not waste what we have*.”
**Call for collective action**	25	“*We need to take care of our environment as much as we can. Natural events contribute to environmental contamination, so we need to mitigate their effect as much as possible by reducing our contribution to the problem*.”
**Positive Environmental Action**	4	“*I think the EPA has prevented much of what we were exposed to a couple of generations ago (or more), but there’s still work to be done. The emissions from vehicles still need to eventually stop or be drastically reduced*.”
**Issues are overstated**	4	“*I feel many people are blowing environment issues too much out of proportion - God is still in control of His creation*”
**Negative health impacts**	13	“*Our air quality seems to be getting worse with every passing year. I have really bad allergies year round. Now it has turned into allergic asthma*”
**Related to agricultural/rural exposures**	5	“*The air, soil, and water are all important in Iowa because we are an agricultural state. My Grandfather used to farm. He is retired now but still leases out his land to be farmed*.”
**Social determinants of health**	3	“*So I stay in Satina, KS and because of my financial struggles at the moment I am experiencing neighbors who pollute indoors the building and it affects my health in a bad way*.”

**Table 3:** Community and Household Disaster Preparedness by Rurality

	Rural (N=62)	Suburban (N=166)	Urban (N=182)	Overall (N=410)
**How prepared is your community for natural disaster?**				
Mean (SD)	2.81 (0.955)	3.16 (0.890)	2.93 (1.08)	3.01 (0.995)
Median [Min, Max]	3.00 [1.00, 5.00]	3.00 [1.00, 5.00]	3.00 [1.00, 5.00]	3.00 [1.00, 5.00]
**How prepared is your household for natural disaster?**				
Mean (SD)	3.05 (1.08)	3.05 (1.03)	2.89 (1.19)	2.98 (1.11)
Median [Min, Max]	3.00 [1.00, 5.00]	3.00 [1.00, 5.00]	3.00 [1.00, 5.00]	3.00 [1.00, 5.00]

## Data Availability

The data presented in this study are available upon request from the corresponding author due to privacy of the survey and focus group participants.
